# The Antifungal Properties of Epidermal Fatty Acid Esters: Insights from White-Nose Syndrome (WNS) in Bats

**DOI:** 10.3390/molecules23081986

**Published:** 2018-08-09

**Authors:** Craig L. Frank, Katherine G. Sitler-Elbel, Anna J. Hudson, Melissa R. Ingala

**Affiliations:** 1 Department of Biological Sciences, Fordham University, Louis Calder Center, Armonk, NY 10504, USA; 2 Environmental Science Program, Fordham University, Bronx, NY 10458, USA; ksitlerelbel@fordham.edu (K.G.S.-E.); ahudson4@fordham.edu (A.J.H.); 3 Richard Gilder Graduate School, The American Museum of Natural History, New York, NY 10024, USA; mingala@amnh.org

**Keywords:** Free Fatty Acid (FFA), antifungal effects, wax ester, 1-monoacylglycerol, epidermis, sebum, *P. destructans*, bats, hibernation, 1,3-diacylglycerol

## Abstract

Numerous free fatty acids (FFAs) are known to have potent antifungal effects. The mammalian epidermis contains both FFAs and multiple classes of fatty acid esters, including 1-monoacylglycerols and wax esters. We thus hypothesized that wax esters and 1-monoacylglycerols composed of antifungal fatty acids would also have antifungal properties. We tested this hypothesis by examining the effects of 1-monoacylglycerols, 1,3-diacylglycerols, and wax esters on the growth of *Pseudogymnoascus destructans* (*Pd*), the fungus that causes White-nose Syndrome (WNS) in North American bats by invading their epidermis. Laboratory experiments with *Pd* cultures demonstrated that: (a) three 1-monoacylglycerols (1-monopalmitolein, 1-monoolein, and 1-monolinolein), as well as, (b) two wax esters, behenyl oleate and behenyl palmitoleate, profoundly inhibit *Pd* growth. The normal growth cycle of *Pd* was interrupted by addition of two cholesterol esters to the media as well. A bat species resistant to cutaneous *Pd* infections has these 1-monoacylglycerols in the epidermis, and another *Pd* resistant bat species has these wax esters in the sebum, thus cutaneous lipid composition is one factor which enables some bats to avoid WNS. Our experiments also revealed that the fatty acid esters which inhibit *Pd* growth are not hydrolyzed by the lipases secreted by this fungus, whereas the esters that do not inhibit *Pd* growth are hydrolyzed.

## 1. Introduction

Several emergent fungal diseases have decimated multiple vertebrate populations during the past 30 years. Each involves a fungal pathogen that invades the epidermis of susceptible vertebrate species. Chytridiomycosis in North and South America is caused by *Batrachochytrium dendrobatidis* and was first observed in 1998. This fungus has since been observed in over 520 amphibian species worldwide, causing severe population declines as well as extinctions in many instances for various infected species [1]. Snake Fungal Disease (SFD) is caused by a severe cutaneous infection with the fungus *Ophidiomyces ophiodiicola* and was first seen in North America in 2006 [2]. This fungus has since been observed infecting the epidermis of 23 snake species, causing severe SFD and mortality in at least 3 of them [3], although one other snake species readily recovers from this cutaneous infection [4].

White-nose Syndrome (WNS) causes mortality rates of 75–98% during hibernation by four bat species (*Myotis lucifugus*, *M. sodalis*, *M. septentrionalis*, *Perimyotis subflavus*) in North America [5]. The fungus *Pseudogymnoascus destructans* (*Pd*) causes WNS, and it grows on the muzzle, wings, and ears of bats during torpor [6]. The *Pd* hyphae penetrate both the epidermis and dermis, causing severe lesions [7]. The optimal temperature for *Pd* growth is 12.5–15.8 °C, and growth is inhibited at temperatures above 19.0 °C [8]. WNS was first found at 6 hibernation sites in central New York State during the winter of 2007–2008. *P. destructans* was introduced from Europe and has since spread to bat hibernation sites located in 33 U.S. states and 7 Canadian provinces [9].

Bouts of torpor during hibernation last for weeks, interrupted by brief (<2 h) periods of euthermy, known as arousal episodes, that account for ~90% of the depot fat utilized [10]. Bats severely infected with *Pd* arouse more frequently from torpor during hibernation, which leads to a premature depletion of depot fat reserves, and subsequent death [11,12]. Big brown bats (*Eptesicus fuscus*) hibernating in mines where *Pd* occurs have torpor bouts of normal duration, and do not develop extensive cutaneous *Pd* infections [13].

The vertebrate epidermis is the first line of defense against cutaneous fungal infections [14]. This leads to the question: What are the epidermal properties that permit some vertebrate species to avoid severe cutaneous fungal infections with a novel pathogen? The epidermis is mostly composed of keratinocytes that occur in 4 distinct strata; they are produced in the stratum basale, which is the deepest layer, and migrate to the stratum corneum with age [15]. The epidermis of many vertebrates contains a complex lipid mixture to reduce cutaneous water loss. Some frog species secrete a mixture of wax esters, cholesterol, triacylglycerols, and free fatty acids (FFAs) from cutaneous glands that form an external film covering their epidermis. The keratinocytes of reptiles produce lipids which are secreted into their intracellular spaces that form a mixture of cholesterol, ceramides, and FFAs [16]. The lipids of the mammalian stratum corneum are a mixture from both the extracellular matrix secreted by keratinocytes, and sebum produced by sebaceous glands. The extracellular matrix produced by keratinocytes contains free sphingosine bases, ceramides, cholesterol, and FFAs, whereas the sebum is composed of triacylglycerols, diacylglycerols, FFAs, wax esters, squalene, cholesterol, and cholesterol esters [17,18]. The epidermis of bats also contains cerebrosides and monoacylglycerols [19,20].

Numerous studies indicate that some of the cutaneous lipids produced by reptiles, mammals, and some frogs prevent fungal infections. Many FFAs are known to have potent antifungal effects [21]. Monoacylglycerols consist of a single fatty acid linked to a glycerol molecule by an ester bond [22], and several have been shown to inhibit fungal growth [23]. Wax esters are composed of a single fatty acid molecule bound to a fatty alcohol by an ester bond [22], and one, pentacosyl heptacosanoate, is a powerful insecticide found in the cuticle of a plant species [24]. The epidermis of *E. fuscus* contains almost twice as much myristic (C14:0), palmitoleic (C16:1Δ9*cis*), and, oleic (C18:1Δ9*cis*) acids as that of *M. lucifugus*. Myristic, palmitoleic, oleic, and linoleic (C18:2Δ9,12*cis,cis*) acids all inhibit *Pd* growth [25]. One of the monoacylglycerols found in the epidermis of *E. fuscus*, 1-oleylglycerol (MG(18:1/0:0/0:0)), 1-monoolein), also greatly inhibits *Pd* growth [26]. The sebum from *Myotis myotis*, another bat species that is highly resistant to cutaneous *Pd* infections, contains over 120 distinct types of wax esters which together account for one-third of this lipid mixture [27].

We thus hypothesized that many of the cutaneous 1-monoacylglycerols and wax esters of vertebrates have antifungal properties. Antifungal lipids disrupt the function of fungal plasma membranes by inserting themselves into the phospholipid bilayers of fungal plasma membranes [28]. For antifungal lipids to insert themselves into fungal plasma membranes, they must be absorbed intact and thus cannot be hydrolyzed by the lipolytic enzymes secreted by fungi into their medium. We thus also hypothesized that the lipid classes that inhibit the growth of a fungal species are not hydrolyzed by the lipases secreted by that species, whereas the lipid classes that are hydrolyzed do not inhibit fungal growth. Laboratory culture experiments with *Pd* maintained on media varying in lipid composition were conducted to test these hypotheses. We also conducted additional culture experiments to determine the effects of sebum diacylglycerols and cholesterol esters on *Pd* growth.

## 2. Results

**Figure 1:**
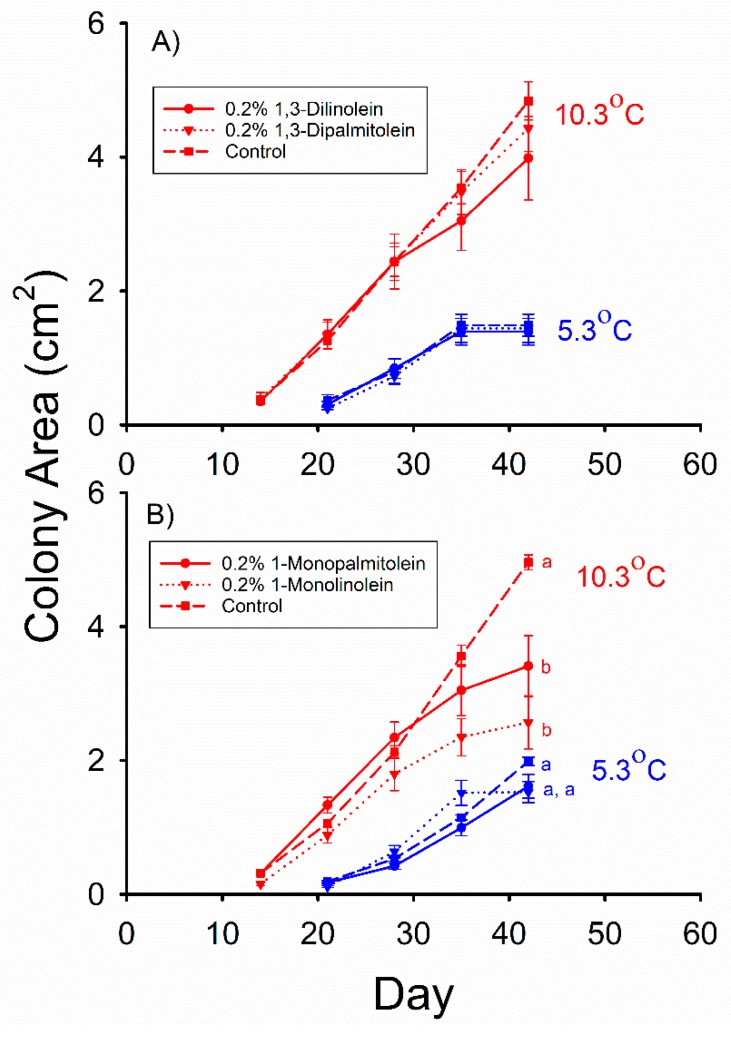
Mean (± SE) surface areas of *Pseudogymnoascus destructans* colonies at various stages of growth in: (**A**) Experiment 1, and (**B**) Experiment 2 at T_a_ = 5.3 (blue symbols) and 10.3 °C (red symbols). Means within the same T_a_ treatment and experiment sharing a common lower-case letter are not significantly different at the *p* < 0.05 level.

All media in Experiments 1 through 5 allowed the production of a normal *Pd* mycelium consisting of mostly hyphae. The control, 0.2% 1,3-dipalmitolein (DG(16:1/0:0/16:1)), and, 0.2% 1,3-dilinolein (DG(18:2/0:0/18:2)) media in Experiment 1 did not significantly differ in the mean *Pd* colony areas observed at either 10.3 °C (F_2,52_ = 1.057, *p* = 0.36) or 5.3 °C (F_2,57_ = 0.055, *p* = 0.95) after being incubated for 42 d (Figure 1A). The mean *Pd* colony areas observed in media containing 0.2% 1-monopalmitolein (MG(16:1/0:0/0:0)) and 0.2% 1-monolinolein (MG(18:2/0:0/0:0)) in Experiment 2 were both less than that observed in the control for this experiment (Figure 1B) after 42 d of incubation at 10.3 °C (F_2,44_ = 14.308, *p* < 0.001). However, the mean *Pd* colony areas observed in these three media did not significantly differ (Figure 1B) after being incubated for 42 d at 5.3 °C (F_2,56_ = 2.899, *p* = 0.063).

**Figure 2:**
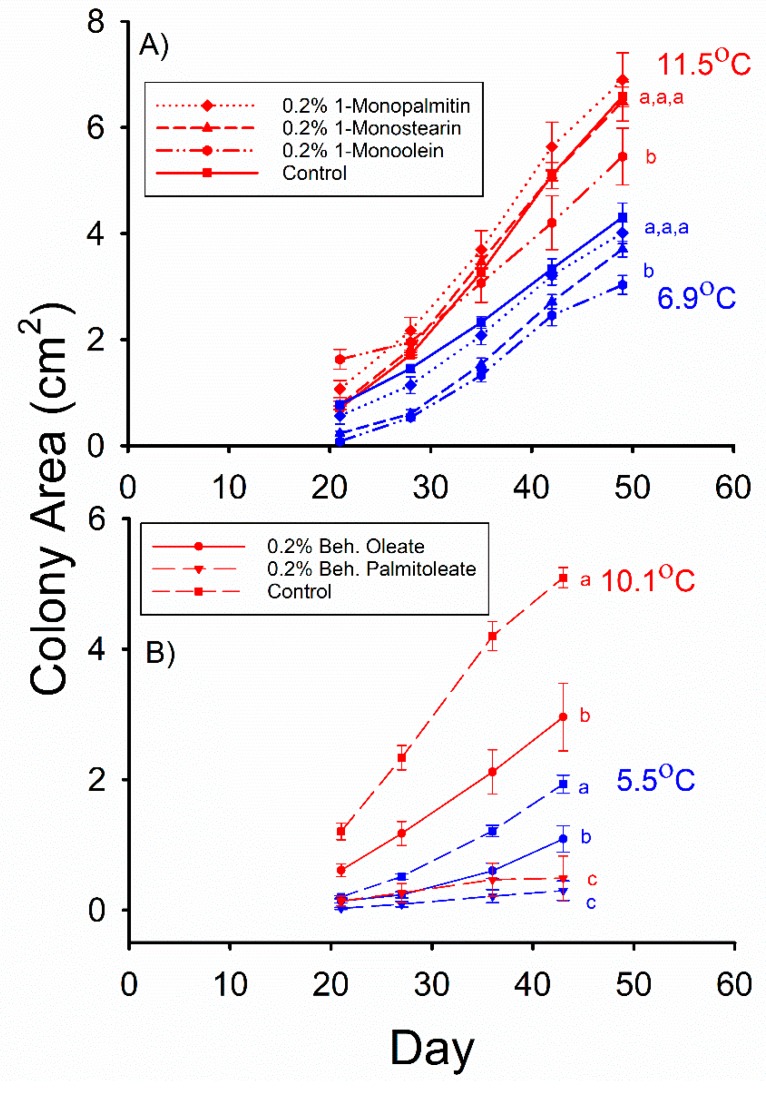
Mean (± SE) surface areas of *Pseudogymnoascus destructans* colonies at various stages of growth in: (**A**) Experiment 3, and, (**B**) Experiment 4 at T_a_ = 5.5–6.4 (blue symbols) and 10.1–11.5 °C (red symbols). Means within the same T_a_ treatment and experiment sharing a common lower-case letter are not significantly different at the *p* < 0.05 level.

The mean *Pd* colony areas observed in the control, 0.2% 1-monopalmitin (MG(16:0/0:0/0:0)), and 0.2% 1-monostearin (MG(18:0/0:0/0:0)) media were not significantly different from each other after 49 d of incubation at 11.5 °C in Experiment 3 (Figure 2A). However, the mean *Pd* colony area observed in the 0.2% 1-monoolein (MG(18:1/0:0/0:0)) medium was less than that observed in the control for this incubation temperature (F_3,66_ = 5.024, *p* = 0.003). The mean *Pd* colony areas observed in the control, 0.2% 1-monopalmitin, and 0.2% 1-monostearin media were not significantly different after 49 d of incubation at 6.9 °C (Figure 2A), and the mean *Pd* colony area observed in the 0.2% 1-monoolein medium was less than that observed in the control for this incubation temperature (F_3,76_ = 7.188, *p* = 0.0003).

**Figure 3:**
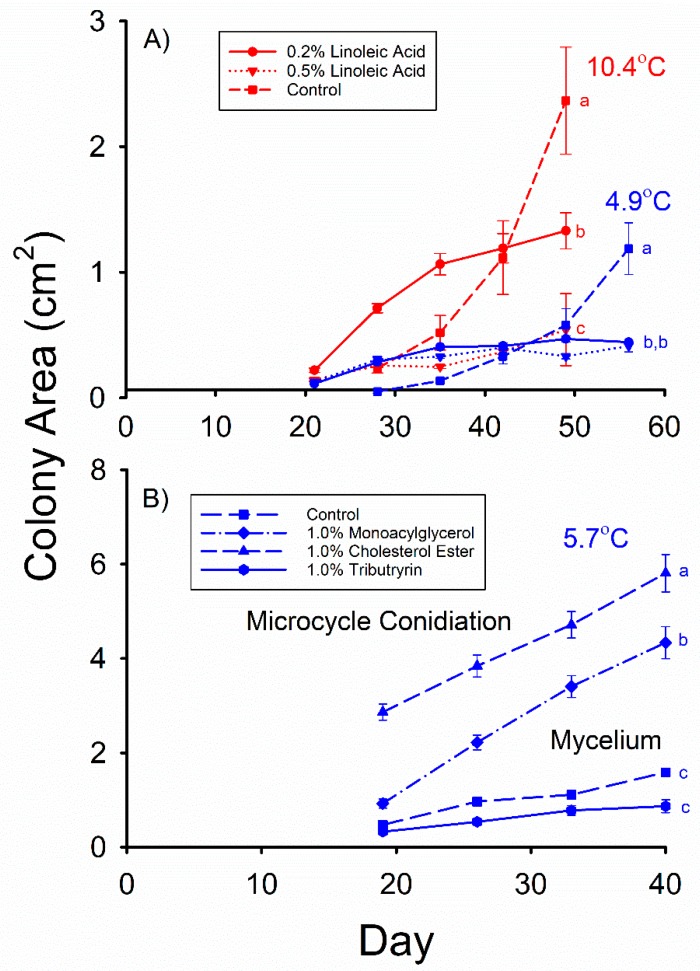
Mean (± SE) surface areas of *Pseudogymnoascus destructans* colonies at various stages of growth in: (**A**) Experiment 5, and (**B**) Experiment 6 at T_a_ = 4.9–5.7 (blue symbols) and 10.4 °C (red symbols). Means within the same T_a_ treatment and experiment sharing a common lower-case letter are not significantly different at the *p* < 0.05 level.

The mean *Pd* colony areas observed in both the 0.2% behenyl palmitoleate (WE(22:0/16:1)) and 0.2% behenyl oleate (WE(22:0/18:1)) media were less than that observed in the control after 43 d of growth at 5.5 °C (Figure 2B) in Experiment 4 (F_2,55_ = 24.593, *p* < 0.001), and the mean *Pd* colony area observed in the 0.2% behenyl palmitoleate medium was also significantly less than that observed in the 0.2% behenyl oleate medium. The same relative relationship among these three media was also observed (Figure 2B) after 43 d of incubation at 10.1 °C (F_2,51_ = 33.382, *p* < 0.001), with the mean *Pd* colony area observed in the 0.2% behenyl oleate medium being 6 times greater than that observed in the 0.2% behenyl palmitoleate medium. The mean colony areas observed in the 0.2 and 0.5% linoleic acid (C18:2Δ9,12*cis,cis*) media were both significantly less than that observed in the control after 49 d of growth at 10.4 °C (Figure 3A) in Experiment 5 (F_2,26_ = 9.910, *p* = 0.001), and the mean *Pd* colony area observed in the 0.2% linoleic acid medium was greater than that observed in the 0.5% linoleic acid medium. At 4.9 °C, the mean colony areas observed in the 0.2% and 0.5% linoleic acid media were both less than that observed in the control (F_2,37_ = 20.33, *p* < 0.001), but they did not significantly differ from each other after 56 d of growth (Figure 3A).

Comparing the results of Experiments 1 through 5 revealed that at 4.9–6.9 °C, 0.2% behenyl palmitoleate inhibited *Pd* growth to the greatest extent relative to control after 42–49 d of incubation (Table 1). Linoleic acid (0.2%) reduced mean *Pd* growth to about one-third of the control, whereas the mean level of *Pd* growth for the 0.2% 1-monoolein and 0.2% behenyl oleate media were each about one half to two-thirds of control levels (F_3,70_ = 11.693, *p* < 0.001) after 42–49 d days at 4.9–6.9 °C (Table 1). The mean *Pd* colony areas observed in the 0.2% linoleic acid, 1-monoolein, 1-monopalmitolein, 1-monolinolein, and behenyl oleate media were all 52–69% of their controls after 42–49 d of incubation at 10.1–11.5 °C (Table 1). The mean *Pd* colony areas observed in the 0.2% behenyl palmitoleate media relative to control was less than one-fifth of this level (Table 1) at 10.1–11.5 °C and was significantly less than those of all other media (F_5,90_ = 8.760, *p* < 0.001).

**Figure 4:**
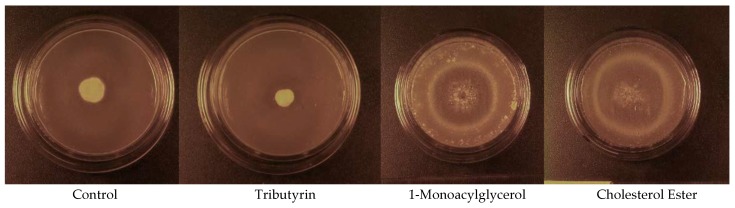
Photographs of *Pseudogymnoascus destructans* colonies after 19 d of growth at 12.7 °C in Experiment 6 on the control, 1.0% tributyrin, 1.0% 1-monoacylglycerol, and 1.0% cholesterol ester media.

The addition of cholesterol esters (CE(16:0) and CE(18:0)) as well as 1-monoacylglycerols (MG(16:0/0:0/0:0) and MG(18:0/0:0/0:0)) to the media in Experiment 6 resulted in microcycle conidiation, whereas a normal mycelium was produced by the control and tributyrin (TG(4:0/4:0/4:0)) media. The cholesterol ester media in Experiment 6 produced a mean *Pd* colony area that was 3.6 times that of the control after 40 d of incubation at 5.7 °C (Figure 3B), whereas the mean *Pd* colony area observed in the 1-monoacylglycerol media was 2.7 times greater than that observed in the control (F_3,71_ = 74.198, *p* < 0.001). The mean *Pd* colony areas observed in the control and 1.0% tributyrin media were not significantly different after 40 d of incubation at 5.7 °C (Figure 3B), however. All the tributyrin plates had clear zones around the *Pd* colonies after 40 d of incubation, indicating lipolysis, whereas no clear zones were observed around the *Pd* colonies on either the cholesterol ester or 1-monoacylglycerol plates. The areas of the *Pd* colonies of some of the media incubated at 12.7 °C grew to the edge of the petri dishes by day 19; thus, this portion of the experiment was terminated at 19 days of incubation. The mean (±SE) *Pd* colony areas observed in the 1.0% cholesterol ester and 1.0% 1-monoacylglycerol media on day 19 were 9.03 ± 0.91 and 7.55 ± 0.77 cm^2^, respectively, and both were significantly greater than that observed in the control (F_3,68_ = 54.187, *p* < 0.001), which was 0.91 ± 0.05 cm^2^. The control and tributyrin media did not significantly differ in the mean *Pd* colony area observed after 19 d of incubation at 12.7 °C, however, and the mean (± SE) *Pd* colony area observed in the tributyrin medium was 0.54 ± 0.07 cm^2^. The *Pd* colonies that grew on the control and tributyrin (Figure 4) media consisted of an extensive mycelium that was 5–7 mm tall by the end of the experiment. In contrast, the *Pd* colonies on the 1-monoacylglycerol and cholesterol ester (Figure 4) media were < 1.0 mm tall and consisted almost entirely of darkly pigmented conidia. These growth patterns occurred at both incubation temperatures.

## 3. Discussion

The results of our study clearly demonstrate that several cutaneous 1-monoacylglycerols containing 16–18 carbon fatty acids which are found in the epidermis of bats inhibit the growth of *Pd*. Mycelium growth at 10.3–11.5 °C was decreased substantially by the addition of 0.2% 1-monoolein, 1-monopalmitolein, and 1-monolinolein to the media, and was also decreased at 6.9 °C by 0.2% 1-monoolein. Microcycle conidiation occurred when the media contained 1.0% 1-monopalmitin plus 1-monostearin as well. However, the level of *Pd* growth inhibition caused by 0.2% 1-monoacylglycerols was less than that exhibited by the addition of 0.2% linoleic acid to the media, however (Table 1).

Our study also reveals that two of the wax esters found in the sebum of *M. myotis*, behenyl oleate and behenyl palmitoleate, inhibit *Pd* growth. This is the first study, to the best of our knowledge, demonstrating that wax esters have antifungal properties. The addition of 0.2% behenyl palmitoleate inhibited *Pd* growth more than the addition of any other lipid type examined, including linoleic acid (Table 1). The sebum wax ester compositions of most bat species are unknown, including those of North American bats. A recent study on *M. myotis* in Europe revealed that the sebum wax esters of this species were composed of fatty alcohols containing up to 35 carbon atoms, and fatty acids composed of up to 32 carbon atoms. All the fatty alcohols were saturated, whereas the fatty acids contained 0 to 4 carbon-carbon double bonds [27]. It would thus be of interest to examine the effects of: (a) fatty acid unsaturation, (b) fatty acid size, and, (c) fatty alcohol size on the ability of a wax ester to inhibit *Pd* growth. Our laboratory is presently conducting experiments examining these wax esters. Unsaturated FFAs tend to have greater antifungal effects than saturated FFAs [28], and the only polyunsaturated FFA found in the epidermis of bats, linoleic acid, inhibits *Pd* growth to a greater extent than the saturated and monounsaturated 14–18 carbon FFAs found in these tissues [25,26]. It is therefore possible that the antifungal activity of wax esters increases with unsaturation in the fatty acid portion of the molecule.

Some of the 1-monoacylglycerols and wax esters found in the epidermis of bats thus have antifungal properties at a physiological concentration (0.2%), and this appears to be one of the factors that enable both *E. fuscus* and *M. myotis* to resist cutaneous infection with *Pd*. The addition of 1,3-dipalmitolein, as well as 1,3-dilinolein, to the media did not affect *Pd* growth. This indicates that the diacylglycerols found in mammalian sebum do not have antifungal properties. A previous study revealed that several triacylglycerols do not inhibit *Pd* growth [26], and 1.0% tributyrin did not affect *Pd* growth in Experiment 6. The triacylglycerols found in mammalian sebum thus do not appear to have antifungal properties as well. Mammalian sebum is on average 45% triacylglycerols, 23–29% wax esters, 10–14% squalene, 10% cholesterol esters, 4% FFAs, and 2% diacylglycerols [29]. Bat sebum is also about 10% 1-monoacylglycerols [20]. The findings of our study therefore indicate that as much as half of the lipids found in the sebum of some bat species may have antifungal activity. We therefore propose that the ability of some bats species to resist *Pd* infection, and thus WNS, is in part due to the wax ester, FFA, and 1-monoacylglycerol composition of their epidermal lipids. It is also possible that the frog species that are resistant to cutaneous infection with *B. dendrobatidis,* and the snake species that survive skin infections with *O. ophiodiicola*, have greater levels of antifungal FFAs, cholesterol ester, and/or wax esters in their epidermis. Determining the effects of specific wax esters, cholesterol esters, and FFAs on the growth of these two fungal species would provide essential information for the prediction and management of both Chytridiomycosis and SFD. A recent study demonstrated that neither host ecology nor phylogeny can predict the susceptibility of a snake species to SFD [3].

About 26% of the amphibian species that declined due to the invasion of Australia by *B. dendrobatidis* are now recovering [30]. Nine of the frog species in Panama that declined due to Chytridiomycosis began to recover just 5–13 years after the first appearance of this disease. Laboratory experiments with two of these frog species demonstrated that their recovery was due to the development of a resistance to cutaneous infection with *B. dendrobatidis*, and not due to the attenuation of this pathogen [31]. The first population of *M. lucifugus* that was infected with *Pd* has since developed a Type II resistance to this fungus [32]. Natural selection should favor the evolution of an epidermal lipid content that provides adequate protection from both cutaneous water loss and fungal invasion. The evolution of a resistance to a cutaneous fungal pathogen by a vertebrate population therefore may in part entail changes in the relative proportions of particular wax ester, 1-monoacylglycerol, and/or FFA types in the epidermis. The addition of 1.0% cholesteryl stearate plus cholesteryl palmitate to media also produced microcycle conidiation, indicating that some cholesterol esters inhibit *Pd* growth. Microcycle conidiation is caused by poor environmental conditions and is a survival mechanism. It is particularly important to pathogens that can infect their hosts only under a limited range of environmental conditions [33]. The potential antifungal properties of the cutaneous cholesterol esters produced by vertebrates therefore warrant further investigation. The tributyrin added to the media in Experiment 6 was hydrolyzed by *Pd*, whereas the 1-monoacylglycerols and cholesterol esters added were not. This supports our hypothesis that the lipids that inhibit the growth of a fungal species are not hydrolyzed by the lipases secreted by that species. The levels of monoacylglycerols in the epidermis of bats recognized as highly susceptible to *Pd* infections, such as *M. lucifugus*, *M. sodalis*, *M. septentrionalis*, and *P. subflavus*, are not known. There also are no published reports on sebum wax ester composition for any bat species found in North America. The wax ester, monoacylglycerol, and free fatty acid contents of the epidermis from *M. lucifugus*, *M. sodalis*, *M. septentrionalis*, *P. subflavus*, as well as *E. fuscus*, therefore must be both measured and compared to fully determine the role of cutaneous lipids in the resistance to *Pd* infection. Further investigation of the role epidermal lipids in the resistance of bat species/populations to cutaneous *Pd* infections will provide new insights to the role of these lipids in other fungal diseases of vertebrates as well.

## 4. Materials and Methods

The *Pd* used in this study is a single strain previously isolated from affected bats in New York State during February 2008 (American Type Culture Collection, Manassas, VA, USA, ATCC MYA-4855). For each experimental media/temperature treatment examined, starter material was transferred using a sterile inoculating needle to a single point in the center of each of 9–22 experimental media plates (petri dishes), yielding 9–22 replicates for every experimental media/temperature combination. The skin temperature (T_skin_) of torpid *M. lucifugus* is normally 5–7 °C during hibernation [10,12], whereas the T_skin_ of torpid *E. fuscus* under similar conditions is 7.5–13.3 °C [13]. Experiments were thus conducted at low (4.9–6.9 °C) and high (10.1–12.7 °C) ambient temperatures to simulate conditions on the skin of torpid *M. lucifugus* and *E. fuscus*. Each group of plates was incubated for 19–49 d. Normal growth was quantified by measuring the total surface area visible for each mycelium. The surface area of each colony (mycelium) was measured at 7 d intervals by capturing digital images of each culture plate with an UVP Chromato-Vue (Upland, CA, USA) model C-75 viewing cabinet. We then calculated the surface area of each photographed colony using ImageJ Version 1.34S software (NIH, Bethesda, MD, USA). Measurements of colony areas started once they were visible to the unaided eye, which was after 12–14 d of incubation. Microcycle conidiation is a fungal stress response to adverse environmental conditions where fungi bypass their normal life cycle and instead asexually form conidia without any mycelial growth [33]. We thus also examined our *Pd* cultures for this response as well. 

Inoculated plates were sealed inside plastic containers with sterile paper towels moistened with sterile water to maintain a relative humidity of ~100% during incubation. A single iButton model DS1922L logger (Maxim Semiconductor, Dallas, TX, USA) was placed inside each container to measure ambient temperature (T_a_) at 1 h intervals throughout incubation. Six different growth experiments were performed, involving 2–3 different types of modified media each, and their compositions are listed in Table 2. The control treatment in all experiments consisted of plates that contained media only.

All treatments in Experiments 1 through 5 consisted of plates containing Sabouraud dextrose agar (SDA). Enough lipids were added to the experimental media to bring their total content to 0.2%, which was within the range of individual 1-monoacylglycerol, wax ester, and fatty acid contents previously reported for the wing epidermis of *M. myotis* and/or *E. fuscus* [20,27]. Experiment 1 was conducted to determine the effects of diacylglycerols on *Pd* growth. Experiments 2 and 3 determined the effects of 1-monoacylglycerols containing either saturated or unsaturated fatty acids that were 16–18 carbon atoms long, which are the types of 1-monoacylglycerols found in the epidermis of *E. fuscus* [20]. Experiment 4 examined the effects of two wax esters found in the sebum of *M. myotis*, behenyl oleate (WE(22:0/18:1)) and behenyl palmitoleate (WE(22:0/16:1)), on *Pd* growth. Linoleic acid has previously been shown to greatly inhibit *Pd* growth at concentrations as low as 0.5%, and to have the greatest inhibitory effects on *Pd* growth of all free fatty acids found in the epidermis of bats [25]. Experiment 5 was thus conducted to determine the relative effects of a 0.2% linoleic acid concentration on *Pd* growth.

All plates in Experiment 6 contained tributyrin agar base media for the detection of lipolysis by *Pd*. The ability to hydrolyze a specific lipid type is determined by adding enough lipid to this media to bring the content to 1.0%, which in turn makes the media opaque. Lipolysis of the lipid in the media by microbial colonies growing on it will render the media transparent, creating clear zones around the colonies in an otherwise turbid medium [34]. The control media in Experiment 6 was tributyrin agar base with no lipid added. Tributyrin (TG(4:0/4:0/4:0)) was added in the second treatment (Table 2) to test for the ability to hydrolyze triacylglycerols. A mixture of 2 cholesterol esters, cholesteryl stearate (CE(18:0)) and cholesteryl palmitate (CE(16:0)) in equal parts, was added to the media in the third treatment to examine for the ability to hydrolyze cholesterol esters. A mixture of two monoacylglycerols, 1-monopalmitin (MG(16:0/0:0/0:0)) and 1-monostearin (MG(18:0/0:0/0:0)) in equal parts, was added to the media in the fourth treatment (Table 2) to determine if *Pd* can hydrolyze 1-monoacylglycerols. All diacylglycerols, monoacylglycerols, as well as wax and cholesterol esters were esters of 16–18 carbon fatty acids found in the epidermis of bats [20,25]. The linoleic acid, tributyrin and cholesterol esters were all >99% pure and obtained from the Sigma-Aldrich Chemical Co. (St. Louis, MO, USA). The monoacylglycerols, diacylglycerols, and wax esters were also >99% pure and produced by Nu-Chek Prep (Elysian, MN, USA). All lipids were added after the media had been autoclaved and cooled to 70–80 °C. A sterile magnetic stirring bar was then immediately added to the flask containing the media, where it stirred continuously at 700 rpm to completely mix the added lipid into the media. All plates were poured while the media was still at a temperature >70 °C, which is above the melting points of all lipid types used in this study, to further ensure that the added lipids were completely mixed into the media.

The *Pd* growth observed in Experiments 1–5 consisted of a normal mycelium in each treatment, and many of the fatty acid esters examined reduced mycelium (colony) area (see Results). The mean % of control colony area was thus calculated for each treatment and the end Experiments 1–5 that significantly affected *Pd* growth, using the mean *Pd* control colony area obtained for the specific experiment in which a particular lipid media was used. This was done so the relative effects of different lipid treatments could be compared between experiments. Mean colony areas at the end of each *Pd* growth experiment were compared between treatments within the same T_a_ group using a one-way ANOVA (General Linear Models) procedure in conjunction with Tukey’s Highly Significant Difference (HSD) Test. All statistical methods were performed using SYSTAT version 13.0 software. Significance level was set at *p* < 0.05 in each case.

## Figures and Tables

**Table 1:** Mean (± SE) % of control colony area for each media treatment that significantly affected *Pd* growth in Experiments 1–5.

Temperature (°C)	Media Composition	% Control Colony Area
4.9–6.9	0.2% Linoleic Acid	37.3 ± 1.2 ^a^^*^
	0.2% 1-Monoolein	70.4 ± 4.1 ^b^
	0.2% Behenyl Oleate	56.5 ± 10.5 ^a^
	0.2% Behenyl Palmitoleate	15.5 ± 7.7 ^c^
10.1–11.5	0.2% Linoleic Acid	65.7 ± 4.4 ^a^
	0.2% 1-Monoolein	82.7 ± 8.1 ^a^
	0.2% 1-Monopalmitolein	68.7 ± 9.2 ^a^
	0.2% 1-Monolinolein	51.8 ± 8.0 ^a^
	0.2% Behenyl Oleate	58.1 ± 10.2 ^a^
	0.2% Behenyl Palmitoleate	9.6 ± 6.7 ^b^

* Means sharing a common lower-case letter within the same temperature category are not significantly different at the *p* < 0.05 level.

**Table 2:** Media, ambient temperature (T_a_), and sample size (N) for the *P. destructans* growth experiments.

Experiment	Media Composition	Low T_a_ (°C)	N	High T_a_ (°C)	N
1	Control	5.3	16	10.3	22
	0.2% DG(16:1/0:0/16:1)	5.3	22	10.3	16
	0.2% DG(18:2/0:0/18:2)	5.3	22	10.3	17
2	Control	5.3	19	10.3	17
	0.2% MG(16:1/0:0/0:0)	5.3	19	10.3	11
	0.2% MG(18:2/0:0/0:0)	5.3	21	10.3	19
3	Control	6.9	20	11.5	20
	0.2% MG(18:1/0:0/0:0)	6.9	20	11.5	18
	0.2% MG(16:0/0:0/0:0)	6.9	20	11.5	15
	0.2% MG(18:0/0:0/0:0)	6.9	20	11.5	18
4	Control	5.5	19	10.1	18
	0.2% WE(22:0/18:1)	5.5	19	10.1	20
	0.2% WE(22:0/16:1)	5.5	20	10.1	16
5	Control	4.9	9	10.4	12
	0.2% C18:2Δ9,12*cis,cis*	4.9	15	10.4	12
	0.5% C18:2Δ9,12*cis,cis*	4.9	16	10.4	9
6	Control	5.7	21	12.7	18
	1.0% TG(4:0/4:0/4:0)	5.7	17	12.7	18
	1.0% Cholesterol Esters	5.7	19	12.7	19
	1.0% 1-Monoacylglycerols	5.7	18	12.7	17
